# Valorization of okra waste: Microencapsulation of okra flower polyphenol‐rich extract with maltodextrin and gum Arabic by freeze drying, spray drying, and microwave drying

**DOI:** 10.1111/1750-3841.70111

**Published:** 2025-03-17

**Authors:** Hojjat Pashazadeh, Ali Ali Redha, Joel B. Johnson, Ilkay Koca

**Affiliations:** ^1^ Department of Gastronomy and Culinary Arts, Faculty of Art and Design Istanbul Nisantası University Istanbul Turkey; ^2^ Hafızbaba Bitkisel ve Kozmetik Ürünler Pazarlama Gıda Sanayi Tic. Ltd. Şti Istanbul Turkey; ^3^ The Department of Public Health and Sport Sciences, Faculty of Health and Life Sciences University of Exeter Medical School Exeter UK; ^4^ Centre for Nutrition and Food Sciences, Queensland Alliance for Agriculture and Food Innovation (QAAFI) The University of Queensland Brisbane Queensland Australia; ^5^ Department of Food Engineering, Faculty of Engineering Ondokuz Mayis University Samsun Turkey

**Keywords:** agriculturalwaste, bioaccessibility, encapsulation, okra, polyphenols

## Abstract

Okra (*Abelmoschus esculentus* (L.) (Moench.)) flowers are under valorized byproducts of okra production. They are a rich source of nutrients and have shown strong antioxidant activity. This study has optimized the extraction of phenolics, flavonoids and antioxidants from okra flowers by response surface methodology. The effect of extraction temperature (26.4–93.6°C), time (6–75 min), and solvent volume (18–102 mL) was evaluated. The optimum extraction conditions were determined as 80°C, solvent volume of 85 mL, and extraction time of 61 min. The extract prepared at optimum conditions was then microencapsulated with maltodextrin, gum Arabic, and a combination of both (1:1) using different drying techniques (freeze drying, spray drying, and microwave drying). The physicochemical properties of the resulting products were analyzed and characterized using differential scanning calorimetry, scanning electron microscopy, and infrared spectroscopy. The drying method impacted the polyphenol levels and bioaccessibility, physical powder properties, and surface characteristics of the resultant product, while the microencapsulation method mainly impacted the polyphenol content, glass transition temperature, and polyphenol bioaccessibility to a lesser extent. Microencapsulation with maltodextrin resulted in powders with significantly higher quercetin‐3‐glucoside (90–100 mg/kg), and epicatechin (480–580 mg/kg) content. The combination of freeze drying with maltodextrin microencapsulation provided the highest content of various polyphenols and bioaccessibility, as well as a very low water activity (0.038 ± 0.000). However, the powder was less free‐flowing compared to microwave drying and tended to have a lower solubility. Consequently, the optimum drying and microencapsulation method will be a compromise between the different importance afforded to each of the aforementioned parameters.

## 1 INTRODUCTION

Okra (*Abelmoschus esculentus* (L.) Moench.)), also known as lady's finger, is a nutrient‐rich tropical vegetable (Erfani Majd et al., 2019). Okra contains various phytochemicals including oligomeric catechins and several flavonol derivatives such as quercetin (Esmaeilzadeh et al., 2020). In vitro and in vivo (animal studies) have shown strong evidence suggesting the anti‐hyperglycemia and anti‐hyperlipidemia activity of different okra products (Nikpayam et al., 2021).

Okra flowers are considered agricultural byproducts and are commonly discarded as waste. However, they have shown strong antioxidant activity associated with their bioactive polysaccharides (Zhang et al., 2020). In addition, okra flowers contain 40.77 ± 0.83 mg gallic acid equivalents (GAE)/g dry weight (DW) of phenolic compounds (Geng et al., 2015). The flavonoid‐rich extract of okra flower has also shown high antioxidant activity and antitumor activity against colorectal cancer through the initiation of mitochondrial dysfunction‐associated apoptosis, senescence, and autophagy (Deng et al., 2020).

Okra flower valorization presents an opportunity to minimize agricultural waste and promote the development of functional food ingredients or dietary supplements for well‐being. These flowers represent a potential source of polyphenols—including phenolic acids and flavonoids—for incorporation into various food products. However, polyphenols are generally heat‐sensitive and susceptible to degradation during food processing by enzymatic or nonenzymatic reactions (Liu et al., 2024). In addition, these compounds can also undergo degradation during digestion upon human consumption due to stomach acid, enzymes, and oxygen. Microencapsulation, a technique where the plant extract is protected by wall materials (e.g., polymers), offers a strategy to overcome these limitations.

Different wall materials have been used for microencapsulating bioactive compounds from natural sources (Dias et al., 2015). Maltodextrin and gum Arabic are two of the commonly used wall materials in microencapsulation. Maltodextrin is used due to its high solubility, low viscosity, thermal stability, and exceptional protective effect against oxidation (Xiao et al., 2022). However, it has a low emulsifying ability; thus, it is usually combined with other polymers such as gum Arabic to improve emulsification. Gum Arabic, also known as acacia gum, has various properties such as pH stability, low viscosity, high solubility, and high emulsifying ability, which makes it a good candidate for microencapsulating plant bioactives (Almayda et al., 2024).

Microencapsulation relies on a critical drying step that significantly impacts the bioactive content, physicochemical properties, size, microstructure, and morphology of the microcapsules (Mehta et al., 2022). Freeze drying and spray drying are the two most common techniques (Rezvankhah et al., 2019). While the low operating temperatures of freeze drying are beneficial for preserving heat‐sensitive phytochemicals, this process is expensive and time‐consuming for large‐scale production. Conversely, spray drying offers a cost‐effective solution for large volumes, but high drying temperatures can degrade unstable phytochemicals (Rezvankhah et al., 2019). Microwave drying could potentially be a compromise as it is economical and requires a shorter drying time compared to spray drying, which can minimize the heat impact on labile components.

As mentioned earlier, the drying technique has a significant influence on the microstructure and morphology of microcapsules, which affects the release of bioactive compounds from the matrix (Jafari et al., 2023; Mardani et al., 2024). Powders formed by freeze drying are porous and have a sponge‐like structure due to the sublimation of ice crystals during the process (Feng et al., 2024). On the other hand, spray drying results in the formation of denser particles with lower porosity compared to freeze drying (Osanlóo et al., 2022). Powders formed by microwave drying can have disrupted internal structures with cracks due to uneven moisture removal during the rapid heating process (Dong et al., 2025). Studies have shown that the release of phenolic compounds from dried fruits such as black grape (Ozkan et al., 2022), cherry (Bayram et al., 2024), and plums (Yener et al., 2024) is affected by the drying method. A study demonstrated that microencapsulated spinach extract, prepared using spray‐ and freeze‐drying methods with proteins, showed significant variations in the release of phenolic compounds and carotenoids, depending on the drying technique employed (Grace et al., 2022). This highlights the importance of further research in this area to advance the production conditions of microcapsules, focusing on selecting suitable drying methods to enhance bioaccessibility.

This research aimed to investigate the valorization of okra waste by producing microencapsulated phenolic‐rich okra flower powder using different drying techniques. Due to limited research on polyphenol extraction from okra flowers, the initial phase focused on optimizing the conditions for extracting phenolics, flavonoids, and antioxidants. Subsequently, the second phase explored the influence of various microencapsulating wall materials (maltodextrin, gum Arabic, and their combination) and drying techniques (freeze drying, spray drying, and microwave drying) on the physicochemical properties and bioaccessibility of the resulting microcapsules.

## 2 MATERIALS AND METHODS

### 2.1 Okra flower sampling and processing

Local agricultural authorities were consulted to identify certified okra‐cultivating farmlands in Samsun, Turkey. Okra flowers were obtained from at least 15 healthy okra plants, with the help of a botanist, from five different organic farms in the Bafra district of Samsun (41.5620° N, 35.9057° E) during the autumn harvest season of 2020. The samples were immediately transferred to the lab and dried in a convective oven (Thermo Scientific™ Heratherm™ Advanced Protocol Oven; Thermo Fisher Scientific Inc.) at 60°C, overnight (∼18 h), until they reached a constant mass with 10% water content. The dried okra flowers were turned into powder using a commercial grinder and passed through a 2‐mm sieve. The dried samples were stored in sealed plastic bags at −18°C during the experimental period.

### 2.2 Optimization of extraction process

The extraction process was optimized by response surface methodology (RSM) following a three‐level central composite design (Design Expert software 13.0). The independent variables considered for optimization were temperature of extraction (°C) as variable A, duration of extraction (min) as variable B, and volume of solvent used for extraction (mL of deionized water) as variable C. The dependent variables were total phenolic content (TPC) in mg/g, total flavonoid content (TFC) in mg/g, antioxidant activity in terms of (i) 2,2‐diphenyl‐1‐picrylhydrazyl (DPPH) reduction in mmol/g and (ii) ferric ion reducing antioxidant power (FRAP) in mmol/g, and individual polyphenols (gallic acid, chlorogenic acid, catechin, epicatechin, and quercetin‐3‐glucoside) in mg/kg. The amount of dry okra flowers was kept constant (2.0 g). Overall, the combination of the independent variables generated 17 experimental runs, and the outcome data were fitted according to the quadratic polynomial model:

(1) $$Y=\beta_{0}+\sum_{i=1}^{k}\beta_{i}X_{i}+\sum_{i=1}^{k}\beta_{ii}X_{ii}+\sum_{i=1}^{k-1}\sum_{i=i+1}^{k}\beta_{ij}X_{i}X_{j}+ɡ$$

where *Y* is the response; *X* is the independent variable; *β*_0_ is the model intercept coefficient; *βi*, *βii*, and *βij* are interactive, quadratic, and linear coefficients; *k* is the number of independent factors; and *Ƹ* is the experimental error. The relationship between independent variables and responses was examined using the analysis of variance (ANOVA) test.

### 2.3 Analysis of total phenolic and flavonoid content

A volume of 1 mL of extract was analyzed for TPC and TFC spectrophotometrically according to the Folin–Ciocalteu assay and aluminum chloride assay, respectively (Kaba et al., 2023). For the TPC, a 20 µL aliquot of extract and 1580 µL of distilled water were mixed with 100 µL of 10% Folin–Ciocalteu reagent and vortexed for 1 min. After 5 min, 300 µL of 7.5% sodium carbonate solution was added, followed by vortexing and a 2‐h incubation in the dark at room temperature. The absorbance of the samples was then measured at 765 nm using a UV/VIS spectrophotometer (LAMBDA™ 365; Perkin Elmer). For the TFC, 1 mL of extract was mixed with 0.3 mL of 5% NaNO_2_ and allowed to react for 5 min. Then, 0.5 mL of 5% AlCl_3_ was added, followed by a 6‐min wait. Next, 0.5 mL of 1 M NaOH was added and vortexed. After 10 min, the absorbance was measured at 510 nm. The TPC and TFC were reported as gallic acid and epicatechin equivalents, respectively.

### 2.4 Analysis of antioxidant activity

A volume of 1 mL of extract was spectrophotometrically analyzed for antioxidant activity following the DPPH and FRAP protocols (Kaba et al., 2023). For the DPPH assay, 50 µL of extract was mixed with 1 mL of DPPH solution (0.06 mM in 80% methanol) and incubated in the dark at room temperature for 1 h. The absorbance was then measured at 517 nm to calculate % DPPH scavenging activity. For the FRAP assay, 50 µL of extract was mixed with 950 µL of FRAP solution (containing 100 mM acetate buffer, 10 mM FeCl_3_, and 10 mM TPTZ). The solution was shaken for 5 min, and the absorbance was measured at 593 nm. The DPPH and FRAP activities were reported as equivalents of Trolox and iron (II) sulfate, respectively.

### 2.5 Analysis of individual polyphenols

The amount of gallic acid, chlorogenic acid, vanillic acid, salicylic acid, catechin, epicatechin, and quercetin‐3‐glucoside was determined using liquid chromatography coupled to a mass spectrometer detector (LC‐MS 8040; Shimadzu) (Pashazadeh et al., 2024). The LC‐MS system was equipped with electrospray ionization (ESI), an autosampler (SIL‐30AC), two pumps (LC‐30 AD), a degassing unit (DGU‐20A 3 R), and a column oven (CTO‐10AS VP). The MS system was operating with the help of an ESI, having the following conditions: 3 L/min nebulizing gas flow rate, 15 L/min drying gas flow rate, 250°C DL temperature, and 400°C heat block temperature. A C18 reversed‐phase column (Inertsil ODS‐4, 3 µm, 4.6 mm × 50 mm; GL Sciences) was used as the stationary phase with a temperature maintained at 30°C. The mobile phase solvents were A—water: formic acid (99.9:0.1 v/v) and B—methanol: formic acid (99.9:0.1 v/v). The solvent flow rate was 0.4 mL/min with a gradient flow in terms of mobile phase A: 0 min, 86% A; 34 min, 63% A; 36 min, 63% A; 51 min, 62% A; 53 min, 62% A; 67 min, 56% A; 69 min, 56% A; 69–90 min, 86% A; 95 min, 86% A. In terms of sample preparation of extract, a volume of 1 mL of extract was diluted with 1 mL of 80% methyl alcohol. In terms of sample preparation of microcapsules, an amount of 0.5 g of powder was dissolved in 10 mL of deionized water. Then, 1 mL of the resulting extract was diluted with 1 mL of 80% methanol and subjected to analysis. The polyphenols of interest were identified based on their retention time and quantified based on peak area. External standards (0–200 mg/L), prepared in methanol, were used for creating the standard curves.

### 2.6 Microencapsulation of okra flower extract

Okra flower extract was prepared at the identified optimum conditions (extraction temperature = 80°C, extraction time = 61 min, and sample to solvent ratio = 2.0 g/85 mL) following the previously described methodology. Okra flower extract was microencapsulated using (i) maltodextrin (M) (4.0–7.0 dextrose equivalent), (ii) gum Arabic (GA), and (iii) a combination of maltodextrin and gum Arabic (MGA). Briefly, an amount of 90 g of aqueous okra flower extract was mixed with 10 g of M or GA, or a combination of 1:1 M and GA (5 g each). The mixture was heated for 1 h at 60°C and then further mixed with a homogenizer (Dispersers T 25 digital ULTRA‐TURRAX; IKA) at 11,000 rpm for 5 min. The samples were then (i) freeze‐dried (FD) to yield FD‐M, FD‐GA, and FD‐MGA; (ii) spray‐dried to yield SD‐M, SD‐GA, and SD‐MGA; and (iii) microwave‐dried to yield MD‐M, MD‐GA, and MD‐MGA. For freeze drying, samples were frozen using liquid nitrogen and then subjected to freeze drying (FD3; Thomas Australia Pty. Ltd.) for 48 h (Papoutsis et al., 2018). For spray drying, samples were spray‐dried using a spray dryer (Mini Spray Dryer B‐290; Buchi) operating with the following conditions: inlet temperature = 125°C, maximum outlet temperature = 55°C, atomization airflow rate = 601 L/h, liquid feed pump rate = 4 mL/min, main drying airflow rate = 38 m^3^/h, feed solution temperature = 70°C, and feed solution volume = 70 mL (Papoutsis et al., 2018). For microwave drying, samples were microwaved for 10 min with a microwave power of 750 W using a commercial microwave oven (Vestel). The amount of gallic acid, chlorogenic acid, vanillic acid, salicylic acid, catechin, epicatechin, and quercetin‐3‐glucoside present in the nine microencapsulated samples was determined according to Section 2.5.

### 2.7 Characterization of microencapsulated samples

#### 2.7.1 Physicochemical properties

The physicochemical properties, dry matter (Pashazadeh et al., 2022), water activity, bulk density, tapped bulk density (Santhalakshmy et al., 2015), Carr's index (Pashazadeh et al., 2022), and solubility (Fazaeli et al., 2012) of all the encapsulated products were determined according to previously reported methods.

#### 2.7.2 Differential scanning calorimetry

The differential scanning calorimeter (DSC 2010; TA Instruments) was calibrated with indium under nitrogen gas flow. The thermograms of the microencapsulated samples were obtained at a scanning temperature range of 10–60°C with a scan rate of 5°C/min (Pashazadeh et al., 2022). The thermogram was used to determine glass transition temperature using Universal 4.0 C. software.

#### 2.7.3 Scanning electron microscopy

The morphology of the microencapsulated samples was imaged using a scanning electron microscope (JSM‐7001F Schottky Emission; JEOL). An amount of each sample was carefully attached to a stainless stub using double sticky tape (Pashazadeh et al., 2022). The assembly was then instantly sputtered with a gold/palladium target (60/40) at 10 nm using a sputter coater. This process operated with argon gas and a plasma current for a duration of 2 min. The images were recorded at different voltages (5 and 10 kV).

#### 2.7.4 Infrared spectroscopy

The infrared spectrum (650–4000 cm^−1^) of the microencapsulated samples was measured using a Fourier‐transform infrared spectrometer (Spectrum Two; Perkin Elmer).

### 2.8 Bioaccessibility of microencapsulated polyphenols

The bioaccessibility of polyphenols (gallic acid, chlorogenic acid, vanillic acid, salicylic acid, catechin, epicatechin, and quercetin‐3‐glucoside) from the different microencapsulated samples was assessed by performing in vitro gastrointestinal digestion. Materials for this experiment were purchased from Sigma‐Aldrich. An amount of 0.5 g of powder was dissolved in 15 mL of deionized water to form a homogeneous solution. A volume of 5 mL of the solution was then mixed with 20 mL of simulated gastric fluid containing 1.5 mL of 3.2 g/L pepsin (400 U/mg). The pH of the solution was adjusted with 1 M HCl to reduce it to 1.7. The sample was then incubated for 2 h at 37°C water bath with continuous shaking. Then, the pH of the solution was adjusted to 7.0 using 1 M NaOH in preparation for the intestinal phase. A volume of 2.5 mL of 4.8 g/L lipase (Type II, 100–650 units/mg protein [using olive oil and 30‐min incubation]), 30–90 units/mg protein (using triacetin), 4 mL of 5 g/L bovine bile salt, and 1 mL of 0.75 M CaCl_2_ were added to the solution. The sample was incubated again for 2 h at 37°C water bath (Fisherbrand™ Isotemp™ Shaking Water Bath; Thermo Fisher Scientific Inc.) with continuous shaking. Upon completion of the intestinal phase, a portion of the sample was centrifuged (5000 × *g*) at 4°C for 15 min. The supernatant was immediately analyzed for polyphenol content by LC‐MS/MS methodology described earlier in Section 2.5. The bioaccessibility of individual polyphenols was calculated according to Equation (2).

(2) $$\mathrm{Bioaccessibility}\;\mathrm{of}\;\mathrm{polyphenol}\;\left(\%\right)=\frac{\mathrm{Concentration}\;\mathrm{of}\;\mathrm{polyphenol}\;\mathrm{in}\;\mathrm{sample}\;\mathrm{after}\;\mathrm{digestion}}{\mathrm{Concentration}\;\mathrm{of}\;\mathrm{polyphenol}\;\mathrm{in}\;\mathrm{sample}\;\mathrm{before}\;\mathrm{digestion}}\times 100$$

### 2.9 Data analysis

MATLAB software (R2016d) was used for modeling purposes. Optimization models and one‐factor graphics were generated using Design‐Expert software 9.0 (Trial version; Stat‐Ease Inc.). The optimization model adequacy was evaluated based on the determined values of the coefficient of determination (*R* ^2^), adjusted coefficient of determination (adj. *R* ^2^), coefficient of variation (CV), and Fisher's test value (*F*‐value). The regression coefficients were considered significant at *p* < 0.05. The desirability function was used to estimate the optimum parameters. All other experiments were performed in triplicate, and the one‐way ANOVA with post hoc Duncan's test (SPSS, version 21) was applied with the significance at *p* < 0.05.

## 3 RESULTS AND DISCUSSION

### 3.1 Extraction optimization

The first stage of this experiment was the optimization of the extraction process, with respect to maximizing the concentration of the major polyphenols present (gallic acid, chlorogenic acid, catechin, epicatechin, and quercetin‐3‐glucoside). As shown in Table 1, the optimization experiment involved 17 different combinations of temperature (ranging from 26.4 to 93.6°C), extraction time (6–75 min), and solvent volume (18–102 mL), while the sample mass was kept at a constant of 2.0 g.

**TABLE 1:** Experimental design including run conditions with respect to the independent variables and responses.

	Independent variables			Responses								
Run	A: Temperature (°C)	B: Time (min)	C: Solvent volume (mL)	TPC (mg/g)	TFC (mg/g)	DPPH (mmol/g)	FRAP (mmol/g)	Gallic acid (mg/kg)	Chlorogenic acid (mg/kg)	Catechin (mg/kg)	Epicatechin (mg/kg)	Quercetin‐3‐glucoside (mg/kg)
1	26.3641	40.5	60	46.34 ± 7.52	5.88 ± 1.25	65.89 ± 2.11	369.90 ± 23.54	151.25 ± 1.01	115.54 ± 2.21	10.24 ± 0.98	2915.52 ± 30.11	152.25 ± 1.56
2	40	20	85	44.62 ± 6.17	10.00 ± 1.18	80.89 ± 6.10	599.79 ± 26.27	253.20 ± 4.34	265.95 ± 3.87	4.08 ± 0.01	3430.84 ± 76.56	176.07 ± 4.55
3	40	20	35	42.87 ± 4.38	7.13 ± 0.54	104.03 ± 3.98	232.39 ± 17.00	200.47 ± 3.88	193.65 ± 0.98	59.59 ± 2.76	2433.18 ± 100.01	191.69 ± 3.43
4	40	61	85	43.75 ± 0.60	8.96 ± 0.10	143.75 ± 10.10	415.72 ± 26.00	257.76 ± 2.67	233.10 ± 2.11	29.39 ± 0.55	3094.74 ± 87.80	193.85 ± 2.90
5	40	61	35	41.57 ± 7.11	7.91 ± 0.06	46.74 ± 4.11	238.14 ± 34.33	130.59 ± 3.55	67.85 ± 0.21	131.56 ± 0.99	1633.61 ± 73.11	117.36 ± 1.78
6	60	6.02325	60	66.78 ± 0.19	11.05 ± 0.10	114.28 ± 2.11	465.85 ± 40.13	328.57 ± 8.77	247.17 ± 2.51	43.91 ± 1.67	3348.52 ± 121.10	238.00 ± 2.76
7	60	40.5	102.045	77.38 ± 4.37	11.76 ± 2.13	110.64 ± 4.65	556.45 ± 15.90	300.04 ± 3.45	260.22 ± 4.13	73.00 ± 2.45	2756.12 ± 80.98	290.12 ± 8.43
8	60	40.5	60	69.02 ± 7.97	10.31 ± 1.21	152.71 ± 5.23	443.36 ± 12.77	184.99 ± 6.11	247.16 ± 3.82	29.30 ± 0.75	2117.99 ± 141.20	158.68 ± 3.52
9	60	40.5	17.9552	25.01 ± 3.73	2.86 ± 0.09	23.41 ± 0.90	233.05 ± 7.96	180.23 ± 2.56	142.31 ± 6.32	72.14 ± 0.87	965.11 ± 21.65	200.69 ± 2.90
10	60	74.9768	60	79.38 ± 5.14	12.52 ± 0.50	145.60 ± 3.21	392.76 ± 13.25	255.74 ± 1.01	228.22 ± 1.32	87.12 ± 1.34	3242.57 ± 154.33	58.37 ± 0.87
11	60	40.5	60	72.66 ± 0.39	9.57 ± 0.20	131.36 ± 6.01	463.97 ± 11.24	209.23 ± 4.22	198.80 ± 4.27	68.82 ± 2.00	2041.65 ± 129.87	71.87 ± 0.10
12	60	40.5	60	66.78 ± 0.79	11.20 ± 0.93	155.56 ± 2.76	478.96 ± 24.35	255.40 ± 1.75	215.65 ± 6.54	87.92 ± 0.50	1840.16 ± 89.56	85.05 ± 1.12
13	80	20	85	86.67 ± 0.28	13.56 ± 0.73	141.73 ± 7.87	495.36 ± 39.41	339.68 ± 5.76	198.37 ± 3.11	381.59 ± 5.67	3399.64 ± 145.56	430.61 ± 4.89
14	80	61	85	93.40 ± 2.49	14.60 ± 2.21	178.03 ± 6.11	493.58 ± 17.77	534.45 ± 8.65	375.53 ± 5.43	510.38 ± 4.45	3741.37 ± 120.87	514.77 ± 11.51
15	80	20	35	30.46 ± 3.33	3.86 ± 0.30	133.78 ± 4.34	328.58 ± 24.16	377.07 ± 7.52	287.98 ± 2.34	307.38 ± 1.80	3075.51 ± 178.80	390.28 ± 6.80
16	80	61	35	38.63 ± 1.98	5.15 ± 0.06	110.67 ± 3.33	354.82 ± 29.89	345.02 ± 6.43	262.68 ± 5.65	329.47 ± 4.76	2668.17 ± 101.34	320.97 ± 8.32
17	93.6359	40.5	60	78.98 ± 3.30	10.97 ± 0.42	166.95 ± 2.56	399.58 ± 16.37	478.31 ± 10.01	342.68 ± 5.43	496.19 ± 1.11	4061.87 ± 163.80	567.88 ± 5.45

*Note*: The individual polyphenols considered for optimization were gallic acid, chlorogenic acid, catechin, epicatechin, and quercetin‐3‐glucoside considering their content in okra flowers is greater than vanillic acid and salicylic acid. Thus, vanillic acid and salicylic acid were not considered for optimization.

A RSM approach was used to predict the responses of specific analytes (TPC, TFC, DPPH, FRAP, gallic acid, chlorogenic acid, catechin, epicatechin, and quercetin‐3‐glucoside) under different extraction conditions. Second‐order polynomial models were fitted to the dataset, and their ANOVA results and regression fit statistics are given in Tables S2 and S3. The reduced second‐order models of different dependent variables were determined (Figure 1) and expressed as:

(3) TPCmgmggg=70.07+9.61A+2.48B+14.86C+2.14AB+13.38AC−0.1242BC−4.44A2−0.7561B2−8.49C2

(4) TFCmgmggg=10.37+0.8593A+0.3333B+2.78C+0.3261AB+1.90AC−0.2585BC−0.7296A2+0.4594B2−1.12C2

(5) $$\begin{array}{ccc} \mathrm{DPPH}\;\left(\mathrm{mmol}/g\right) & = & 145.54+26.27A+5.23B+21.67C\\ & & +\;0.9533AB+0.1799AC+22.44BC\\ & & -\;7.20A^{2}-2.42B^{2}-24.66C^{2} \end{array}$$

(6) $$\begin{array}{ccc} \mathrm{FRAP}\;\left(\mathrm{mmol}/g\right) & = & 462.39+17.30A-20.27B+102.10C\\ & & +\;25.35AB-29.93AC-27.23BC\\ & & -\;28.36A^{2}-12.60B^{2}-24.82C^{2} \end{array}$$

(7) $$\begin{array}{ccc} \mathrm{Gallic}\;\mathrm{acid}\;\left(\mathrm{mg}/\mathrm{kg}\right) & = & 215.35+95.50A-1.84B+39.06C\\ & & +\;28.50AB-3.48AC+37.66BC\\ & & +\;38.82A^{2}+30.83B^{2}+12.43C^{2} \end{array}$$

(8) $$\begin{array}{ccc} \mathrm{Chlorogenic}\;\mathrm{acid}\;\left(\mathrm{mg}/\mathrm{kg}\right) & = & 219.71+54.62A-2.83B\\ & & +\;33.62C+38.81AB\\ & & -\;26.79AC+36.93BC\\ & & +\;5.88A^{2}+8.91B^{2}\\ & & -\;3.97C^{2} \end{array}$$

(9) $$\begin{array}{ccc} \mathrm{Catechin}\;\left(\mathrm{mg}/\mathrm{kg}\right) & = & 56.56+155.34A+23.49B+7.24C\\ & & +\;6.70AB+51.60AC+7.50BC\\ & & +\;86.38A^{2}+20.02B^{2}+22.51C^{2} \end{array}$$

(10) $$\begin{array}{ccc} \mathrm{Epicatechin}\;\left(\mathrm{mg}/\mathrm{kg}\right) & = & 1999.96+309.02A-101.01B\\ & & +\;502.91C+133.76AB\\ & & -\;132.68AC+151.57BC\\ & & +\;526.25A^{2}+457.96B^{2}\\ & & -\;49.36C^{2} \end{array}$$

(11) $$\begin{array}{ccc} \mathrm{Quercetin} & - & 3-\mathrm{glucoside}\;\left(\mathrm{mg}/\mathrm{kg}\right)=103.15+122.77A\\ & & -\;25.18B+32.62C+8.93AB+21.66AC\\ & & +\;30.70BC+97.18A^{2}+22.27B^{2}\\ & & +\;56.65C^{2} \end{array}$$

FIGURE 1Estimated response surface plots of (a) total phenolic content (TPC), (b) total flavonoid content (TFC), (c) 2,2‐diphenyl‐1‐picrylhydrazyl (DPPH) scavenging activity, (d) ferric reducing antioxidant power (FRAP), (e) gallic acid content, (f) chlorogenic acid content, (g) catechin content, (h) epicatechin content, and (i) quercetin‐3‐glucoside content versa (1) temperature and time, (2) temperature and solvent volume, and (3) time and solvent volume.

**Figure:**
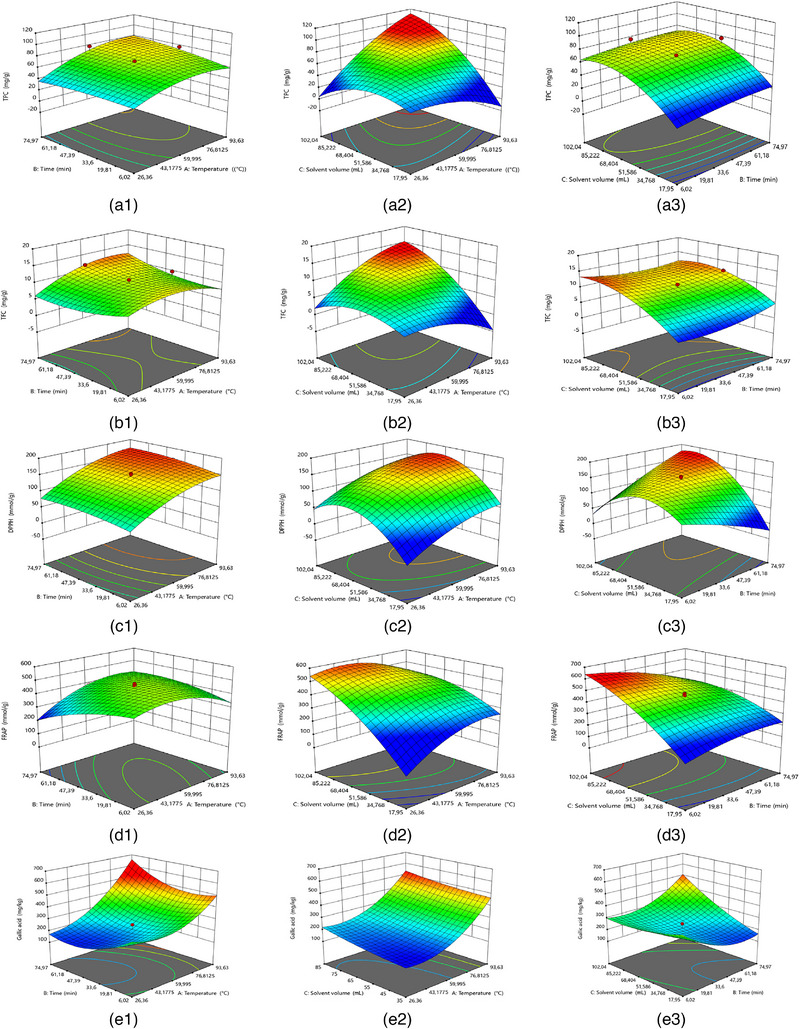

**Figure:**
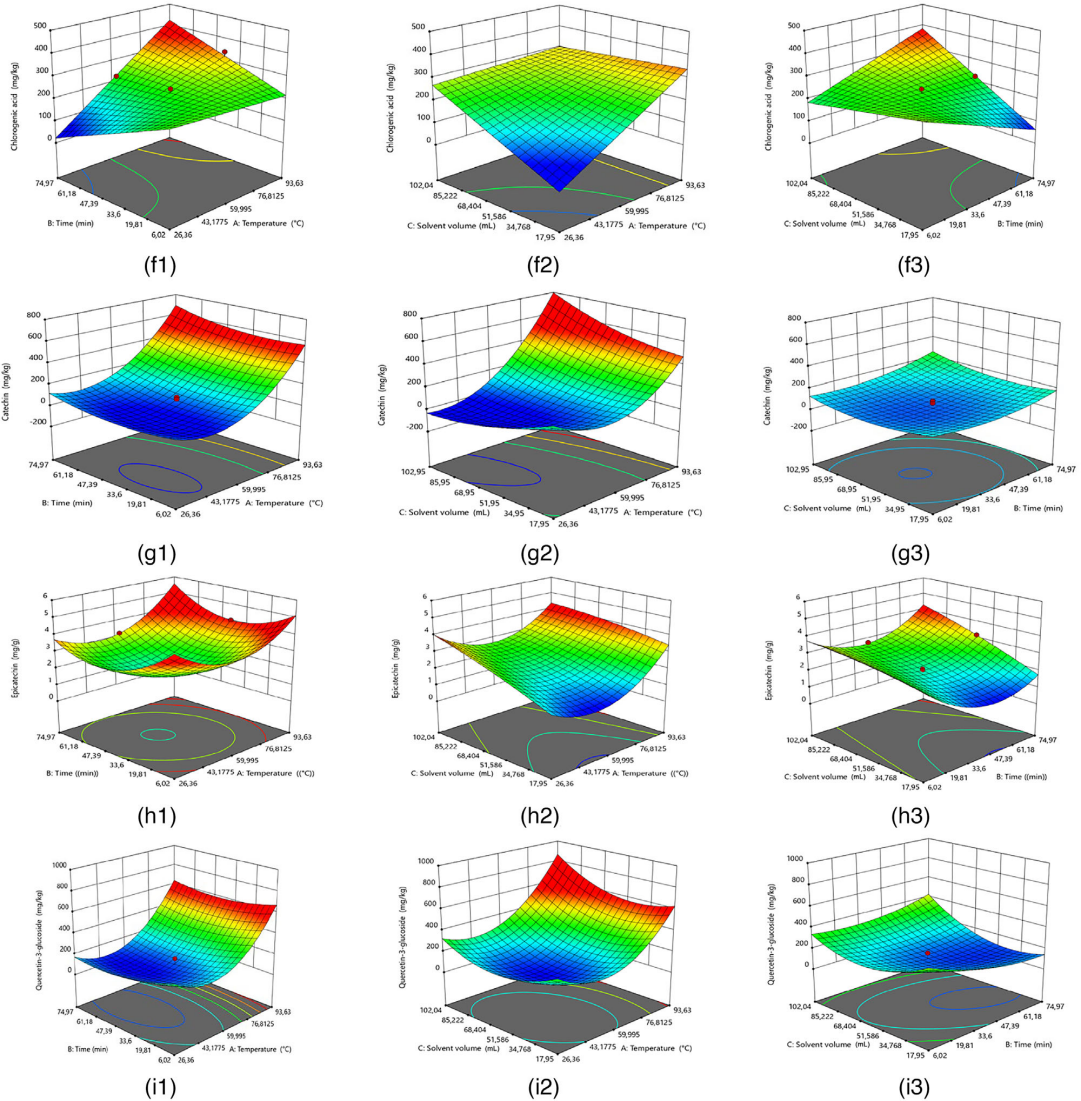

All of the regression models showed a significant fit (*p *< 0.003 for all) and quite high adjusted *R* ^2^ values (*R* ^2^_adj_ of 0.85–0.97). This indicates that they were able to predict the response of the analytes from the independent variables (extraction temperature, time, and volume) with a high level of accuracy. This was also supported by the low coefficients of variation (CV) for these analytes, most of which ranged from 4.7% for epicatechin to 19.6% for quercetin‐3‐glucoside. However, the model for catechin showed a CV of 41.1%, indicating a high amount of variability (or error) associated with its predictive results. This model also showed the lowest adjusted *R* ^2^ and predictive *R* ^2^ values.

The extraction temperature (independent variable A) had a significant impact (*p *< 0.05) on all analytes, while the solvent volume (variable C) also significantly impacted the levels of all analytes except catechin (Figure 1). Antony & Farid (2022) noted that the highest total polyphenol content was usually obtained at an extraction temperature of 60–80°C, although high temperatures could cause degradation. Similarly, the highest TPC was found at 80°C in this study. Notably, the extraction time (variable B) only had a significant impact on the FRAP and epicatechin concentrations. It is likely that the FRAP is influenced by other, non‐polyphenol compounds, such as ascorbic acid and other antioxidants (Demircan et al., 2024), and thus would be influenced by different extraction conditions. A number of analytes (particularly FRAP, chlorogenic acid, and epicatechin) also showed significant interactions between some or all of these independent variables (A, B, and C).

The optimum extraction conditions selected by the RSM were a temperature of 80°C, solvent volume of 85 mL and extraction time of 61 min. These gave predicted concentrations of 98.7 mg/g for TPC and 14.93 mg/g for TFC. Similarly, the predicted antioxidant activities based on DPPH and FRAP were 188 and 464 mmol/g, respectively. In terms of individual polyphenols, a concentration of 493 mg/kg of gallic acid, 365 mg/kg of chlorogenic acid, 437 mg/kg of catechin, 3798 mg/kg of epicatechin, and 471 mg/kg of quercetin‐3‐glucoside were determined. Overall, the desirability statistic for the optimized extraction conditions was found to be 0.889, indicating a good compromise between maximizing the concentration of each of the analytes considered (Boateng, 2023), without markedly decreasing the concentration of the other analytes.

### 3.2 Microencapsulation

Table 2 shows the individual polyphenol contents for each combination of drying technique (freeze drying, spray drying, and microwave drying) and microencapsulation technique (maltodextrin, gum Arabic, and maltodextrin/gum Arabic). The most abundant polyphenol was epicatechin (with concentrations ranging from 137 to 547 mg/kg, depending on processing technique), followed by quercetin‐3‐glucoside (5–102 mg/kg) and catechin (31–92 mg/kg).

**TABLE 2:** Amount of polyphenols (phenolic acids and flavonoids) in microencapsulated okra extract.

	Content (mg/kg)						
Samples	Gallic acid	Chlorogenic acid	Vanillic acid	Salicylic acid	Catechin	Epicatechin	Quercetin‐3‐glucoside
**FD‐M**	52.14 ± 0.09^a^	20.99 ± 0.74^a^	6.94 ± 0.11^a^	39.85 ± 0.02^a^	74.14 ± 0.78^b^	546.71 ± 6.04^a^	100.82 ± 4.15^a^
**FD‐GA**	35.78 ± 1.52^cd^	12.68 ± 0.18^bc^	6.68 ± 0.66^ab^	25.25 ± 1.23^c^	91.98 ± 6.78^a^	252.44 ± 2.46^e^	4.83 ± 0.50^e^
**FD‐MGA**	26.06 ± 5.42^ef^	16.54 ± 8.79^abc^	6.53 ± 0.23^ab^	34.99 ± 0.04^b^	75.47 ± 3.99^b^	227.13 ± 3.00^f^	6.90 ± 1.10^e^
**SD‐M**	42.09 ± 2.98^bc^	12.15 ± 0.03^bc^	6.14 ± 0.24^bc^	15.23 ± 0.50^d^	75.09 ± 0.16^b^	514.14 ± 11.67^b^	101.88 ± 4.43^a^
**SD‐GA**	43.68 ± 6.98^b^	16.92 ± 0.68^abc^	5.79 ± 0.02 ^cd^	8.82 ± 0.11^e^	60.86 ± 4.58^c^	136.56 ± 6.15^g^	5.06 ± 0.09^e^
**SD‐MGA**	32.08 ± 0.50^ce^	15.36 ± 0.61^abc^	5.04 ± 0.03^e^	39.84 ± 0.47^a^	54.49 ± 2.91^c^	247.56 ± 11.20^e^	7.02 ± 0.95^e^
**MD‐M**	17.06 ± 0.20^g^	9.61 ± 0.28^c^	5.46 ± 0.04^de^	38.62 ± 1.11^a^	30.54 ± 0.32^d^	484.45 ± 13.61^c^	91.44 ± 4.85^b^
**MD‐GA**	21.62 ± 0.12^fg^	13.85 ± 0.23^abc^	6.86 ± 0.19^b^	14.55 ± 0.16^d^	87.87 ± 1.97^a^	152.99 ± 8.47^g^	20.32 ± 1.51^d^
**MD‐MGA**	23.01 ± 2.18^fg^	19.19 ± 0.45^ab^	5.61 ± 0.11^cde^	39.27 ± 0.22^a^	35.37 ± 0.60^d^	360.06 ± 3.33^d^	36.24 ± 2.95^c^

*Note*: Data reported as means (*n* = 3) with different superscripts in the same row differ significantly (*p* < 0.05).

Abbreviations: drying techniques: FD, freeze dried; SD, spray dried; MD, microwave dried; wall material: M, maltodextrin; GA, gum Arabic; MGA, maltodextrin and gum Arabic.

Previous studies on this species have reported the main polyphenols as chlorogenic acid, *p*‐coumaric acid and catechin (Agregán et al., 2023). Most of the other polyphenols quantified here (gallic acid, vanillic acid, epicatechin, and quercetin‐3‐glucoside) have all been previously reported (Abdel‐Razek et al., 2023; Arapitsas, 2008).

In general, freeze drying gave statistically higher polyphenol contents compared to SD and MD. This concurred with literature from other species, which supports the proposition that freeze drying provides higher polyphenol contents than other drying methods, such as spray drying (Laureanti et al., 2023), oven drying, or vacuum drying (Demircan et al., 2024; Guebebia et al., 2023; Tan et al., 2020). Similarly, Pashazadeh et al. (2025) found the highest anthocyanin content and bioaccessibility in okra flower extract microcapsules prepared using freeze drying.

Specifically, the highest level of all polyphenols (with the exception of catechin) across all drying and microencapsulation techniques was observed for the combination of freeze drying with maltodextrin microencapsulation. The encapsulation technique also had an effect, with GA and MGA methods almost consistently performing worse than maltodextrin alone. The notable exceptions to this were for chlorogenic acid and salicylic acid, where MGA encapsulation retained significantly higher levels of these phenolic acids compared to maltodextrin encapsulation when paired with microwave drying (for both) and spray drying (for chlorogenic acid). Notably, both of these compounds are moderately sized phenolic acids, whereas the remaining polyphenols are either simple phenolic acids (gallic acid) or flavonoids. Nevertheless, the optimum method for retaining high levels of most polyphenols was the combination of freeze drying and maltodextrin microencapsulation. Similar to the results seen here, Tonon et al. (2010) found that maltodextrin was also the best encapsulation method for providing a high antioxidant activity in spray‐dried açai. However, other researchers have proposed that an MGA combination may provide better protection of polyphenols in certain matrices (Laureanti et al., 2023; Tolun et al., 2016).

### 3.3 Characterization

#### 3.3.1 Physicochemical properties

As well as considering the polyphenol content of the microencapsulated powders, it is crucial to consider their physicochemical properties, as these can impact the powders’ suitability for various pharmaceutical applications or downstream processing techniques. The physical characterization results of the microencapsulated okra extract powder—including the bulk density, tapped bulk density, Carr's index, solubility, water activity, and dry matter content—are given in Table 3.

**TABLE 3:** Physical properties of microencapsulated okra extract powder using different wall materials and techniques.

Samples	Bulk density (g/cm^3^)	Tapped bulk density (g/cm^3^)	Carr's index (%)	Solubility (%)	Water activity	Dry matter (%)
**FD‐M**	0.1760 ± 0.00^d^	0.2479 ± 0.00^d^	29.00 ± 1.41^c^	80.90 ± 1.97^c^	0.0383 ± 0.00^d^	96.60 ± 0.98^ab^
**FD‐GA**	0.1445 ± 0.00^e^	0.1926 ± 0.00^e^	25.00 ± 0.00^d^	100.00 ± 0.00^a^	0.0451 ± 0.00^d^	97.85 ± 1.20^ab^
**FD‐MGA**	0.1546 ± 0.00d^e^	0.2148 ± 0.02^e^	28.00 ± 0.00^c^	91.20 ± 1.31^b^	0.0424 ± 0.00^d^	98.85 ± 0.90^a^
**SD‐M**	0.2243 ± 0.00^c^	0.4042 ± 0.01^c^	44.50 ± 0.50^b^	99.90 ± 0.14^a^	0.2013 ± 0.00^a^	95.91 ± 0.80^ab^
**SD‐GA**	0.2329 ± 0.00^c^	0.4197 ± 0.00^bc^	44.50 ± 0.50^b^	98.40 ± 0.14^a^	0.0972 ± 0.00^bcd^	98.45 ± 0.40^ab^
**SD‐MGA**	0.2291 ± 0.03^c^	0.4482 ± 0.03^b^	49.00 ± 1.00^a^	99.40 ± 0.56^a^	0.1475 ± 0.00^ab^	96.62 ± 1.96^ab^
**MD‐M**	0.5315 ± 0.01^b^	0.6859 ± 0.01^a^	22.50 ± 0.50^e^	99.90 ± 0.00^a^	0.0671 ± 0.00^cd^	98.02 ± 0.00^ab^
**MD‐GA**	0.6029 ± 0.00^a^	0.6851 ± 0.00^a^	12.00 ± 0.00^f^	100.00 ± 0.00^a^	0.1352 ± 0.00^abc^	96.41 ± 0.45^ab^
**MD‐MGA**	0.5884 ± 0.00^a^	0.6764 ± 0.01^a^	13.00 ± 1.41^f^	99.95 ± 0.07^a^	0.0803 ± 0.03^bcd^	95.83 ± 0.03^b^

*Note*: Data reported as means (*n* = 3) with different superscripts in the same row differ significantly (*p* < 0.05).

Abbreviations: drying techniques: FD, freeze dried; SD, spray dried; MD, microwave dried; wall material: M, maltodextrin; GA, gum Arabic; MGA, maltodextrin and gum Arabic.

The bulk density and tapped bulk density were strongly correlated (*r*_7_ = 0.949), with the tapped bulk density being an average of 38% higher than the bulk density for the freeze‐dried powder, 85% higher for spray drying, and 19% higher for microwave drying. The density was lowest for the freeze‐dried powder, followed by the spray‐dried powder. Similarly, Iombor et al. (2014) found a lower density of freeze‐dried soursop powder compared to oven drying. The spray‐dried densities were slightly lower than those reported for spray‐dried acai powder (Tonon et al., 2010), which may be attributed to differences in their composition. Density is related to the molecular weight of the cell wall materials, with heavier materials allowing closer packing and higher bulk densities (Tonon et al., 2010). However, similar to previous studies, there was generally little difference in bulk density between different microencapsulation methods (Akhavan Mahdavi et al., 2016; Tonon et al., 2010). Powder bulk density is an important factor in food processing and can influence flowability (lower density powders often have a poorer flow characteristic).

The Carr's index was lowest for the microwave‐dried powder (12–22.5) and highest among the spray‐dried powder samples (index of 44.5–49). This metric is a measure of the compressibility of a powder, with lower values indicating more free‐flowing powders. However, it is worth noting that while the most dense powders were more free‐flowing (i.e., had a higher Carr's index), the least free‐flowing powders were the spray‐dried powders with a moderate density. Consequently, the microwave‐dried powder would appear to be the most suitable formulation for processing purposes (Mahdi et al., 2020), followed by the freeze‐dried powder.

Most samples showed a high level of solubility (>98%), with only FD‐M and FD‐MGA showing lower solubilities (81% and 91%, respectively). According to de Barros Fernandes et al. (2014), powders with low solubility can result in processing difficulties and cause significant economic losses. The high solubility found here for okra flower extract was also superior to that reported for many other microencapsulated extracts (Laureanti et al., 2023; Mahdi et al., 2020). Thus, the majority of powder formulations reported here would appear to be quite suitable for processing purposes—taking into account their powder bulk density, Carr's indices, and solubility.

The freeze‐dried powders consistently showed a very low level of water activity (0.038–0.045), while the water activity was moderately higher in the microwave‐dried (0.07–0.14) and spray‐dried powders (0.10–0.20). Conversely, the latter samples tended to have a lower dry matter content (i.e., higher moisture content) compared to the freeze‐dried powder. Nevertheless, the water activity of all samples was well below the 0.60 limit required to inhibit microbial growth (Clemenson et al., 2012; Fontana Jr., 2020). This confirms that all of the drying methods investigated here may produce a dried powder suitable to use in pharmaceutical products or similar applications. A lower water activity is also important for maintaining the stability and storage properties of the microencapsulated product (Wang et al., 2020).

The microencapsulation method did not appear to have any consistent, statistically significant impact on any of the physical parameters, including density, solubility, water content/activity, and Carr's index.

Finally, the glass transition temperatures are shown in Table 4. This is the temperature at which a polymeric substance changes from a hard, brittle (“glassy”) state to a viscous or rubbery state. It should be well above the storage temperature to ensure the stability of the product (Sharayei et al., 2020). Higher molecular weight encapsulants can increase the glass transition temperature (Tonon et al., 2010). The transition temperatures were lowest for the gum Arabic and maltodextrin/gum Arabic encapsulants, as well as maltodextrin in combination with freeze drying or spray drying. However, maltodextrin in combination with microwave drying gave the highest glass transition temperature (176°C) of all combinations tested. This was also the combination with the highest tapped bulk density and the lowest water activity (aside from the freeze‐dried samples). This illustrated a high degree of interaction between the microencapsulant and drying method.

**TABLE 4:** Glass transition temperature of microencapsulated okra extract powder using different wall materials and techniques.

Samples	Glass transition temperature (°C)		
	Onset	Peak	End
**M**	156.47 ± 0.67	158.15 ± 0.11	164.69 ± 1.11
**GA**	136.67 ± 1.00	138.74 ± 0.67	146.08 ± 0.89
**MGA**	140.80 ± 0.55	144.07 ± 1.22	150.15 ± 0.97
**FD‐M**	143.40 ± 0.75	144.77 ± 0.76	148.67 ± 1.12
**FD‐GA**	153.70 ± 1.04	155.18 ± 1.90	159.41 ± 0.54
**FD‐MGA**	163.75 ± 1.21	165.02 ± 0.89	168.57 ± 0.85
**SD‐M**	141.39 ± 0.89	143.5 ± 0.45	148.55 ± 0.05
**SD‐GA**	162.68 ± 1.43	164.54 ± 1.45	169.34 ± 1.76
**SD‐MGA**	151.48 ± 0.95	154.01 ± 0.01	161.08 ± 0.67
**MD‐M**	174.65 ± 0.49	176.26 ± 0.52	181.16 ± 1.00
**MD‐GA**	151.75 ± 1.12	153.27 ± 0.67	158.41 ± 0.87
**MD‐MGA**	154.79 ± 0.08	156.59 ± 0.88	162.02 ± 0.76

Abbreviations: drying techniques: FD, freeze dried; SD, spray dried; MD, microwave dried; wall material: M, maltodextrin; GA, gum Arabic; MGA, maltodextrin and gum Arabic.

#### 3.3.2 Surface properties

**FIGURE 2:**
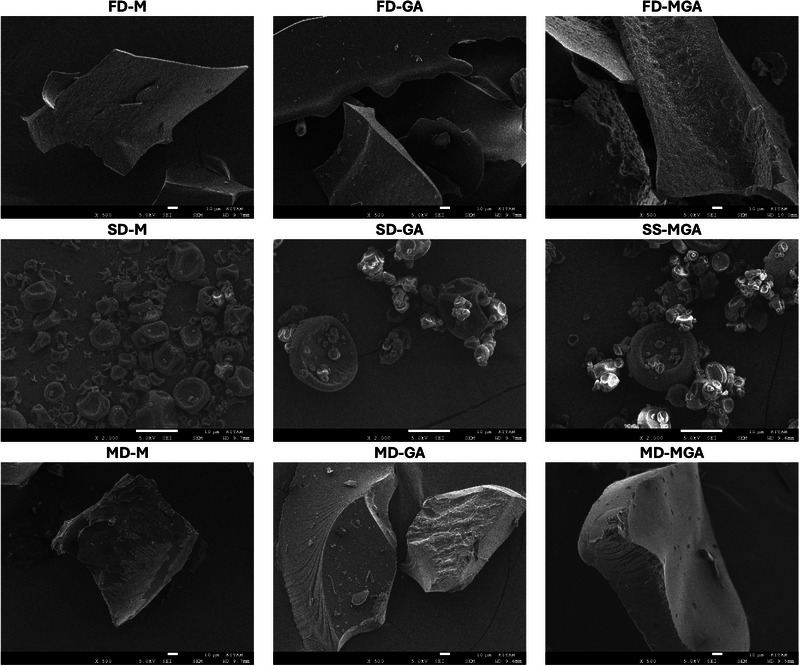
Scanning electron microscopy images of okra microencapsulated samples. Drying techniques: FD, freeze dried; SD, spray dried; MD, microwave dried; wall material: M, maltodextrin; GA, gum Arabic; MGA, maltodextrin and gum Arabic.

Scanning electron microscopy was used to investigate the surface characteristics of the powder produced using each of the drying and microencapsulation combinations. As seen in Figure 2, freeze drying produced larger fragments of sample, which tended to be relatively thin and bore prominent edges. On the other hand, spray drying produced quite small, spherical‐like fragments, similar to observations from previous researchers (Guebebia et al., 2023). This would correspond to the moderate bulk density of these samples (as smaller fragments allow closer packing), while the thin fragments of freeze‐dried material would have more air between them and result in a lower density. On the other hand, the thin freeze‐dried “sheets” better allow for the evaporation of water, likely providing the lower water activity values. Microwave drying also produced large fragments, although these were typically thicker than the corresponding freeze‐dried fragments. This would be responsible for the much higher density of the microwave‐dried samples (Table 3), as there would be more mass of the material and less air between fragments.

**FIGURE 3:**
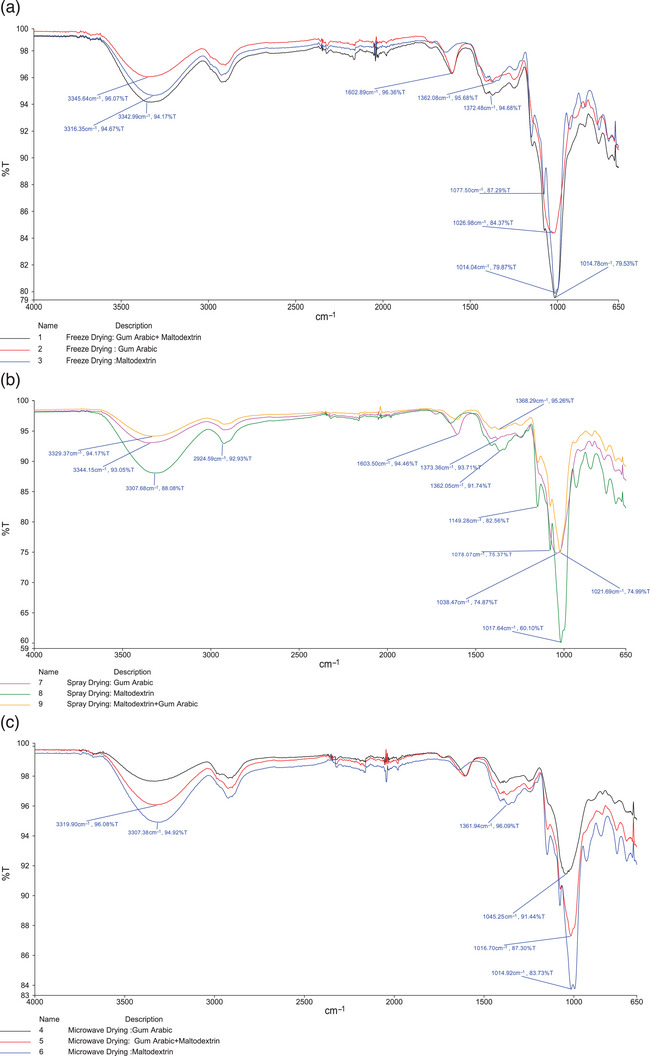
Infrared spectra of microencapsulated okra powder by (a) freeze drying, (b) spray drying, and (c) microwave drying.

Infrared (IR) spectroscopy was used to characterize the functional groups present in each sample and highlight the impact of different microencapsulation methods on the chemical composition (Figure 3). The freeze‐dried samples generally showed similar IR spectra, although the maltodextrin encapsulation resulted in a significantly smaller absorption peak at 1603 cm^−1^, likely attributable to C = O stretch from amides, aromatics, or similar functional groups. The gum Arabic‐encapsulated sample also showed lower absorbance in the 1014 cm^−1^ region. This peak is in the “fingerprint” region of the IR spectrum and is thus difficult to assign to a specific chemical bond. Overall, the IR spectra were also quite similar for the spray‐dried and microwave‐dried powders, indicating similar chemical compositions. Within the spray‐dried group, the maltodextrin encapsulation method showed a much higher absorbance around 1017 cm^−1^, as well as an additional peak at 1149 cm^−1^. As previously mentioned, the former peak is in the fingerprint region, while the latter absorbance may arise from the C–O group of esters. The structure of the maltodextrin monomer contains an ester bond, so it may be responsible for this C–O signal. Within the microwave‐dried powders, the spectra were largely similar aside from some differences in the magnitude of the peaks, particularly around the 1014 cm^−1^ region. Again, the maltodextrin sample was the only sample with a notable peak around 1150 cm^−1^, mirroring the observations made for the spray‐dried powder sample.

### 3.4 Bioaccessibility

Finally, the bioaccessibility of the major polyphenols present in the samples was assessed. As shown in Table 5, the bioaccessibility varied widely for different polyphenols, from as low as 5%–16% for epicatechin to over 100% for vanillic acid. The increase in vanillic acid concentration (i.e., >100%) at the end of the digestion process could be due to the breakdown of larger polymer polyphenols such as lignin or transformation from vanillin (by oxidation) or ferulic acid (by demethylation with side‐chain cleavage) (Lubbers et al., 2021). In general, the most bioaccessible polyphenols were vanillic acid, salicylic acid, and chlorogenic acid. Both catechin and epicatechin had very low bioaccessibilities (9%–22% and 5%–16%, respectively).

**TABLE 5:** Bioaccessibility of phenolic acids and flavonoids from microencapsulated okra powder.

	Bioaccessibility (%)						
Samples	Gallic acid	Chlorogenic acid	Vanillic acid	Salicylic acid	Catechin	Epicatechin	Quercetin‐3‐glucoside
**FD‐M**	46.53 ± 0.12^a^	61.13 ± 1.08^a^	144.50 ± 3.04^abc^	85.51 ± 1.83^a^	22.49 ± 0.21^a^	16.07 ± 0.61^a^	94.24 ± 11.05^a^
**FD‐GA**	44.61 ± 1.80^a^	63.46 ± 1.48^a^	150.87 ± 14.16^ab^	85.00 ± 0.37^a^	14.41 ± 1.03^d^	15.16 ± 0.09^ab^	97.42 ± 11.44^a^
**FD‐MGA**	33.06 ± 0.21^bc^	54.17 ± 6.05^ab^	132.59 ± 4.58^c^	60.18 ± 3.86^d^	9.06 ± 0.71^f^	15.52 ± 0.26^a^	109.19 ± 17.76^a^
**SD‐M**	22.79 ± 1.66^d^	61.51 ± 0.67^a^	140.49 ± 5.59^bc^	65.81 ± 0.02^c^	12.30 ± 0.18^e^	5.12 ± 0.14^e^	8.01 ± 0.23^b^
**SD‐GA**	22.76 ± 3.77^d^	48.76 ± 1.28^ab^	142.39 ± 1.84^bc^	76.03 ± 0.01^b^	16.08 ± 1.03^c^	11.77 ± 0.51^c^	114.32 ± 4.37^a^
**SD‐MGA**	31.05 ± 0.52^c^	44.23 ± 2.32^ab^	156.38 ± 1.67^a^	80.94 ± 1.15^ab^	19.11 ± 0.06^b^	10.52 ± 0.59^d^	113.27 ± 10.32^a^
**MD‐M**	33.64 ± 0.34^bc^	58.23 ± 1.73b^a^	92.28 ± 0.85^e^	37.92 ± 4.35^e^	13.71 ± 0.16^d^	5.46 ± 0.17^e^	4.76 ± 0.52^b^
**MD‐GA**	34.40 ± 3.09^bc^	56.90 ± 0.68^ab^	115.86 ± 1.53^d^	57.30 ± 0.97^d^	13.80 ± 0.37^d^	14.00 ± 1.23^b^	20.57 ± 2.28^b^
**MD‐MGA**	37.61 ± 3.38^b^	35.28 ± 0.52^b^	91.64 ± 1.06^e^	35.85 ± 1.67^e^	16.83 ± 0.06^c^	7.92 ± 0.10^e^	24.22 ± 3.37^b^

*Note*: Data reported as means (*n* = 3) with different superscripts in the same row differ significantly (*p* < 0.05).

Abbreviations: drying techniques: FD, freeze dried; SD, spray dried; MD, microwave dried; wall material: M, maltodextrin; GA, gum Arabic; MGA, maltodextrin and gum Arabic.

While there were some exceptions for specific compounds, the best polyphenol bioaccessibilities were found for the freeze drying and maltodextrin combination, followed by freeze drying and gum Arabic. These combinations generally provided the highest bioavailability for all polyphenols except quercetin‐3‐glucoside and catechin. Notably, the freeze‐dried powder also showed the highest polyphenol contents (Table 2) and typically showed favorable physical properties (Table 3). The improved bioaccessibility of polyphenols in this matrix may be due to the thin, sheet‐like structure of the particles (Figure 2), which would allow the digestive enzymes to better access the material surface.

Consequently, the combination of freeze drying with maltodextrin or gum Arabic microencapsulation appears to be optimal for processing okra flower extract for pharmaceutical applications. Pashazadeh et al. (2025) also noted the highest bioaccessibility for anthocyanins from freeze‐dried microencapsulated okra flower extract. The bioaccessibility of the polyphenols found here was quite promising compared to other literature results, such as 28% bioaccessibility for microencapsulated polyphenols from green tea (Rodrigues Silva et al., 2021).

On the other hand, the microwave‐dried samples consistently showed much lower bioaccessibilities, particularly for vanillic acid, salicylic acid, quercetin‐3‐glucoside, and epicatechin. This is likely due to the much larger particle size (Figure 2) and higher bulk density of these matrices, making it more difficult for the digestive enzymes to access all the bound polyphenols present in the material. The spray‐dried powder samples typically showed moderate polyphenol bioavailability, aside from the maltodextrin combination—which had a low polyphenol bioavailability.

## 4 CONCLUSIONS

Extraction of phenolic, flavonoids, and antioxidants from okra flowers was optimized by response surface methodology. The drying method had a strong influence on polyphenol levels and bioaccessibility, as well as the physical powder properties and surface characteristics of the resultant product. On the other hand, the microencapsulation method mainly impacted the polyphenol content, bioaccessibility, and glass transition temperature, with less influence on other physical powder properties. The most favorable powder properties were afforded using microwave drying, with a higher density and lower Carr's index indicating a free‐flowing powder. However, the highest polyphenol contents and bioaccessibility were found using freeze drying in combination with maltodextrin microencapsulation. This was likely due to the physical structure of these matrices (thin, sheet‐like fragments), with the increased surface area allowing the polyphenols present to readily interact with digestive enzymes and dissolve in the extraction solvent. This polyphenol‐rich powder holds significant potential as an ingredient for developing functional foods that promote health and well‐being in society due to its high antioxidant content. Additionally, this approach supports the sustainable utilization of agricultural waste. However, further research is needed to evaluate the storage stability of the microcapsules under varying temperature and humidity conditions over time to determine potential changes in bioactive content and water activity. Future studies should also explore the incorporation of this powder into food matrices and assess its bioaccessibility and antioxidant potential.

## AUTHOR CONTRIBUTIONS

**Hojjat Pashazadeh**: Conceptualization; investigation; methodology;  validation; visualization; formal analysis; data curation; writing—review and editing. **Ali Ali Redha**: Validation; visualization; formal analysis; data curation; writing—original draft; writing—review and editing. **Joel B. Johnson**: Writing—original draft; writing—review and editing; validation. **Ilkay Koca**: Supervision; project administration; resources.

## CONFLICT OF INTEREST STATEMENT

The authors declare no conflicts of interest.

## Supporting information




**Table A**: Coded and actual values of independent variables.
**Table B1**: Results of ANOVA of the reduced models of total phenolics and flavonoids, and antioxidant activity.
**Table B2**: Results of ANOVA of the reduced models of polyphenols.

## Data Availability

Data are available within the article or its Supporting Information.
