# Supplementary material for: Valorization of okra waste: Microencapsulation of okra flower polyphenol‐rich extract with maltodextrin and gum Arabic by freeze drying, spray drying, and microwave drying
Source: J Food Sci. 2025 Mar 17;90(3):e70111. doi: 10.1111/1750-3841.70111 (PMC11911960; doi:10.1111/1750-3841.70111)
Supplement: Supplementary file 1 — Table A: Coded and actual values of independent variables. Table B1: Results of ANOVA of the reduced models of total phenolics and flavonoids, and antioxidant activity. Table B2: Results of ANOVA of the reduced models of polyphenols. [file JFDS-90-0-s001.docx]

**Supplementary File**

**Valorization of okra waste: microencapsulation of okra flower polyphenol-rich extract with maltodextrin and gum arabic by freeze-drying, spray-drying and microwave-drying**

**Table A:** Coded and actual values of independent variables.

| **Coded values** | **Actual values** | | |
| --- | --- | --- | --- |
|  | A | B | C |
| -1.68 | 26.36 | 6.02 | 17.95 |
| -1 | 40 | 20 | 35 |
| 0 | 60 | 40.5 | 60 |
| 1 | 80 | 61 | 85 |
| 1.68 | 93.63 | 74.97 | 102.045 |

**Table B1:** Results of ANOVA of the reduced models of total phenolics and flavonoids, and antioxidant activity.

|  | **TPC** | | | **TFC** | | | **DPPH** | | | **FRAP** | | |
| --- | --- | --- | --- | --- | --- | --- | --- | --- | --- | --- | --- | --- |
|  | SS | F- value | p-value | SS | F- value | p-value | SS | F- value | p-value | SS | F- value | p-value |
| **Model** | 6754.72 | 17.29 | 0.0005 | 174.16 | 21.87 | 0.0003 | 27410.84 | 10.37 | 0.0028 | 1.827E+05 | 28.23 | 0.0001 |
| **A** | 1261.46 | 29.05 | 0.0010 | 10.08 | 11.40 | 0.0118 | 9424.89 | 32.09 | 0.0008 | 4085.20 | 5.68 | 0.0486 |
| **B** | 84.23 | 1.94 | 0.2063 | 1.52 | 1.71 | 0.2317 | 373.53 | 1.27 | 0.2966 | 5609.03 | 7.80 | 0.0268 |
| **C** | 3017.64 | 69.50 | < 0.0001 | 105.84 | 119.63 | < 0.0001 | 6410.95 | 21.83 | 0.0023 | 1.424E+05 | 197.96 | < 0.0001 |
| **AB** | 36.52 | 0.8412 | 0.3896 | 0.8510 | 0.9618 | 0.3594 | 7.27 | 0.0248 | 0.8794 | 5139.71 | 7.15 | 0.0318 |
| **AC** | 1432.91 | 33.00 | 0.0007 | 29.02 | 32.80 | 0.0007 | 0.2590 | 0.0009 | 0.9771 | 7166.14 | 9.96 | 0.0160 |
| **BC** | 0.1233 | 0.0028 | 0.9590 | 0.5345 | 0.6041 | 0.4625 | 4029.90 | 13.72 | 0.0076 | 5931.40 | 8.25 | 0.0239 |
| **A^2^** | 222.25 | 5.12 | 0.0581 | 6.00 | 6.78 | 0.0352 | 584.55 | 1.99 | 0.2012 | 9066.54 | 12.61 | 0.0093 |
| **B^2^** | 6.44 | 0.1484 | 0.7115 | 2.38 | 2.69 | 0.1450 | 66.02 | 0.2248 | 0.6498 | 1790.58 | 2.49 | 0.1586 |
| **C^2^** | 813.09 | 18.73 | 0.0034 | 14.22 | 16.07 | 0.0051 | 6857.90 | 23.35 | 0.0019 | 6944.53 | 9.66 | 0.0171 |
| **Residual** | 303.92 |  |  | 6.19 |  |  | 2055.77 |  |  | 5034.28 |  |  |
| **Lack of Fit** | 286.31 | 6.50 | 0.1387 | 4.87 | 1.47 | 0.4517 | 1705.93 | 1.95 | 0.3727 | 4395.23 | 2.75 | 0.2878 |
| **Total** | 7058.64 |  |  |  |  | 180.35 | 29466.61 |  |  | 1.877E+05 |  |  |
| **R^2^** | 0.9569 |  |  |  |  | 0.9657 | 0.9302 |  |  | 0.9732 |  |  |
| **Adj R^2^** | 0.9016 |  |  |  |  | 0.9215 | 0.8405 |  |  | 0.9387 |  |  |
| **Pred R^2^** | 0.6881 |  |  |  |  | 0.7756 | 0.5102 |  |  | 0.7870 |  |  |
| **Adeq Precision** | 15.3722 |  |  |  |  | 17.2080 | 11.3103 |  |  | 17.6832 |  |  |
| **C.V. %** | 11.15 |  |  |  |  | 10.17 | 14.52 |  |  | 6.55 |  |  |

**Table B2:** Results of ANOVA of the reduced models of polyphenols.

|  | **Gallic acid** | | | **Chlorogenic acid** | | | **Catechin** | | | **Epicatechin** | | | **Quercetin-3-glucoside** | | |
| --- | --- | --- | --- | --- | --- | --- | --- | --- | --- | --- | --- | --- | --- | --- | --- |
|  | SS | F- value | p-value | SS | F- value | p-value | SS | F- value | p-value | SS | F- value | p-value | SS | F- value | p-value |
| **Model** | 1.852E+05 | 13.74 | 0.0011 | 86720.08 | 13.68 | 0.0012 | 4.444E+05 | 11.43 | 0.0020 | 1.059E+07 | 70.88 | < 0.0001 | 3.59E+05 | 17.31 | 0.0005 |
| **A** | 1.246E+05 | 83.19 | < 0.0001 | 40748.28 | 57.83 | 0.0001 | 3.296E+05 | 76.27 | < 0.0001 | 1.304E+06 | 78.52 | < 0.0001 | 2.06E+05 | 89.29 | < 0.0001 |
| **B** | 46.11 | 0.0308 | 0.8657 | 109.43 | 0.1553 | 0.7052 | 7537.26 | 1.74 | 0.2281 | 1.393E+05 | 8.39 | 0.0231 | 8655.61 | 3.75 | 0.0939 |
| **C** | 20837.33 | 13.92 | 0.0074 | 15433.58 | 21.90 | 0.0023 | 716.00 | 0.1657 | 0.6961 | 3.454E+06 | 207.98 | < 0.0001 | 14527.69 | 6.3 | 0.0404 |
| **AB** | 6499.98 | 4.34 | 0.0757 | 12051.32 | 17.10 | 0.0044 | 359.22 | 0.0831 | 0.7814 | 1.431E+05 | 8.62 | 0.0218 | 637.45 | 0.2765 | 0.6153 |
| **AC** | 97.09 | 0.0649 | 0.8063 | 5741.66 | 8.15 | 0.0245 | 21299.68 | 4.93 | 0.0618 | 1.408E+05 | 8.48 | 0.0226 | 3752.65 | 1.63 | 0.2427 |
| **BC** | 11344.28 | 7.58 | 0.0284 | 10908.42 | 15.48 | 0.0056 | 450.48 | 0.1043 | 0.7562 | 1.838E+05 | 11.07 | 0.0127 | 7538.56 | 3.27 | 0.1135 |
| **A^2^** | 16992.17 | 11.35 | 0.0119 | 389.47 | 0.5528 | 0.4814 | 84116.33 | 19.47 | 0.0031 | 3.122E+06 | 187.98 | < 0.0001 | 1.07E+05 | 46.18 | 0.0003 |
| **B^2^** | 10712.58 | 7.16 | 0.0318 | 895.50 | 1.27 | 0.2967 | 4517.44 | 1.05 | 0.3406 | 2.364E+06 | 142.36 | < 0.0001 | 5593.18 | 2.43 | 0.1633 |
| **C^2^** | 1742.74 | 1.16 | 0.3164 | 177.38 | 0.2517 | 0.6312 | 5713.85 | 1.32 | 0.2879 | 27467.61 | 1.65 | 0.2393 | 36175.17 | 15.69 | 0.0055 |
| **Residual** | 10480.32 |  |  | 4932.17 |  |  | 30244.58 |  |  | 1.163E+05 |  |  | 16138.43 |  |  |
| **Lack of Fit** | 7921.17 | 1.24 | 0.5034 | 3726.83 | 1.24 | 0.5037 | 28456.51 | 6.37 | 0.1413 | 75051.24 | 0.7285 | 0.6652 | 11761.41 | 1.07 | 0.5466 |
| **Total** | 1.956E+05 |  |  | 91652.25 |  |  | 4.747E+05 |  |  | 1.071E+07 |  |  | 3.75E+05 |  |  |
| **R^2^** | 0.9464 |  |  | 0.9462 |  |  | 0.9363 |  |  | 0.9891 |  |  | 0.957 |  |  |
| **Adj R^2^** | 0.8776 |  |  | 0.8770 |  |  | 0.8544 |  |  | 0.9752 |  |  | 0.9017 |  |  |
| **Pred R^2^** | 0.6398 |  |  | 0.6360 |  |  | 0.5289 |  |  | 0.9366 |  |  | 0.7342 |  |  |
| **Adeq Precision** | 13.5273 |  |  | 16.1092 |  |  | 11.9145 |  |  | 30.2868 |  |  | 13.6798 |  |  |
| **C.V. %** | 13.76 |  |  | 11.62 |  |  | 41.05 |  |  | 4.68 |  |  | 19.63 |  |  |
